# Development of high-throughput screening viral titration assay: Proof of concept through two surrogate viruses of human pathogens

**DOI:** 10.1093/biomethods/bpaf049

**Published:** 2025-06-17

**Authors:** Valentin Job, Laura Bonil, Damien Coupeau, Sébastien Penninckx, Emna El Golli-Bennour, Margot Cardinal, Benoit Muylkens, Stéphane Lucas

**Affiliations:** LARN Laboratory (LARN-NARILIS/NISM), University of Namur, Namur, B-5000, Belgium; Namur Research Institute for Life Sciences (NARILIS), Integrated Veterinary Research Unit (URVI), Université de Namur, Namur, 5000, Belgium; Namur Research Institute for Life Sciences (NARILIS), Integrated Veterinary Research Unit (URVI), Université de Namur, Namur, 5000, Belgium; Medical Physics Department, Institut Jules Bordet, Université Libre de Bruxelles, Brussels, B-1000, Belgium; LARN Laboratory (LARN-NARILIS/NISM), University of Namur, Namur, B-5000, Belgium; LARN Laboratory (LARN-NARILIS/NISM), University of Namur, Namur, B-5000, Belgium; Namur Research Institute for Life Sciences (NARILIS), Integrated Veterinary Research Unit (URVI), Université de Namur, Namur, 5000, Belgium; LARN Laboratory (LARN-NARILIS/NISM), University of Namur, Namur, B-5000, Belgium; Innovative Coating Solutions (ICS), University of Namur, Forville, B-5380, Belgium

**Keywords:** viruses, titration, MTS, colorimetric assay, drug screening, high-throughput screening

## Abstract

The precise determination of viral titers in virological studies is a critical step to assess the infectious viral concentration of a sample. Although conventional titration methods, such as endpoint dilution or plaque forming units are the gold standards, their widespread use for screening experiments remains limited due to the time-consuming aspect and resource-intensive requirements. This study introduces a rapid and user-friendly high-throughput screening assay for evaluating viral titers. The colorimetric method used relies upon assessing virus-induced cytopathic effects by measuring the reduction of a tetrazolium reagent to formazan through cellular dehydrogenation within mitochondria. The resulting formazan quantity is correlated with the viral titer and can be easily quantified by a colorimetric measurement. In this perspective, this manuscript describes two case studies for the titration of the porcine respiratory coronavirus virus and bovine alpha herpesvirus 1, highlighting, respectively, a linear regime between 100 and 2000 TCID_50_/ml and 500–$10^{6}$ PFU/ml for rapid titration within these ranges. The proposed technique’s advantages and drawbacks are discussed, along with potential applications such as drug screening and the assessment of viral survival on inert surfaces.

## Introduction

Viral titration is a crucial technique in virology research that quantifies via biological assays the titer of a virus in a given sample. The gold-standard methods are the plaque assay (PFA), the endpoint dilution (ED) assays, e.g. the 50% tissue culture infectious dose (TCID_50_), and the immunofluorescence (IF) foci assay gathered in the group 1 of cell-based assays on Table S1 in the Supplementary information. Other methods are used to quantify viral concentration either indirectly (Group 2) or directly (Group 3). Accurate determination of viral titers is fundamental for several applications, including vaccine development, antiviral drug and materials screening, viral pathogenesis studies and quality control in biopharmaceutical manufacturing processes.

The plaque-forming unit (PFU) assay involves diluting and inoculating a lytic virus onto a semiconfluent monolayer of permissive cells and then restricting the virus propagation within the mechanical flow of the supernatant using immobilizing overlays, allowing the virus to form plaques (dead-cell clearings in the cell monolayer), which are then counted to quantify the viral titer. Not all viruses have the capacity to produce these plaques. The IF assay utilizes virus-specific antibodies and specific fluorescent dyes to visualize and enumerate individual infected cell foci, providing a more sensitive means of quantification. The TCID_50_ method involves seeding cells at a known density and infecting them with serial dilutions of the viral sample. Subsequent optical microscopy observation of cytopathic effects (CPEs) provides a quantal result, wherein each well is scored either positively or negatively based on the presence or absence of CPEs, estimating the dilution that causes CPE in 50% of the cell cultures [1]. The TCID_50_ value can be estimated using various mathematical approaches, including the Reed-Muench method [2, 3] or the Spearman-Kaerber method [4]. The selection of a particular method can be decided by suitable assay criteria, within the constraints of the virus and cell type.

Despite their widespread use and reliability, these methods suffer from several limitations. They are notoriously time-consuming, often requiring 5–12 days to obtain results, hindering rapid decision-making in time-sensitive applications. Moreover, they involve laborious serial dilutions, limiting the number of samples that can be analyzed simultaneously and making the process labor-intensive, especially when dealing with a large number of samples. Subjective interpretation of results, particularly in the case of plaque- or foci-enumeration, or, the dependency on an expert naked-eye observation for CPE effect, introduces potential variability and operator bias, impacting the accuracy and reproducibility of the measurements [5]. Additionally, these techniques may not be suitable for quantifying certain types of viruses that do not form distinct plaques, foci, or CPE, thereby limiting their applicability across diverse viral species. Although these techniques provide a quantitative measurement of the viral titer, the obtained TCID_50_ value is not a direct measure of the quantity of infectious viral particles. It instead reflects the infectivity dose of a viral solution obtained after a calculation. The binary nature when reading each cell monolayer contained in one well incubated with one dilution, infected or not, does not allow this method to correlate the CPE as a quantitative phenomenon to the virus’ infectivity against cells. These drawbacks highlight the need for alternative methods that can overcome these limitations and provide more rapid, objective, and broadly applicable quantification of viral titers.

As an alternative to this ED assay, we have developed a high-throughput screening (HTS) assay [6], using a tetrazolium-based colorimetric approach to assess directly the metabolic activity of infected cells [7, 8]. Tetrazolium dyes, such as [3-(4,5-dimethylthiazol-2-yl)-5-(3-carboxymethoxyphenyl)-2-(4-sulfophenyl)-2H-tetrazolium inner salt (MTS), are converted into formazan by mitochondrial succinate dehydrogenase (Promega Corporation). The absorbance measurement of this solution is directly correlated to cellular metabolic activity. Given that a dead cell is no longer metabolically active, MTS assay enables users to quantify indirectly the extent of viral infection. The main advantages of this ED technique lie in (i) its rapidity, (ii) the fact that no dilution is required when working within the linear range of the technique, which makes the test easy to use, and (iii) providing continuous data on the rate of infected cells, in contrast with the TCID_50,_ which provides discrete data.

While this tetrazolium approach is widely used to evaluate cell proliferation [7, 9, 10], cytotoxicity of different drugs [11–13], the detection of HIV replication inhibitors [14] and biocompatibility of nanomaterial [15–17], this method is rarely applied to the virology field. In addition, HTS methods are required when very large numbers of samples must be analyzed in parallel in various applications. Evaluating the antiviral activity of pharmaceutical drugs [14, 18], nanomaterials such as nanoparticles [17] or antimicrobial coatings [19, 20] are potential applications where the methodology described in this paper could significantly help their development.

The aim of this study was to develop and validate a colorimetric assay that enabled rapid, cost-effective, and objective quantification of viral titers across diverse viral species. Specifically, we investigated a user-friendly HTS assay that indirectly quantifies infectious viruses by assessing the metabolic activity of infected cells using a modified colorimetric MTS assay. We hypothesized that this proposed method would overcome the limitations of conventional viral titration techniques, while maintaining high sensitivity and specificity comparable to other methods.

This approach’s feasibility is shown through a case study quantifying porcine respiratory coronavirus (PRCV) and bovine alpha herpesvirus 1 (BoHV-1) by measuring the metabolic activity of swine testicular (ST) cells and Madin-Darby bovine kidney (MDBK) cells exposed to different viral doses. To validate and compare this HTS assay with conventional methods, the MTS assay is performed alongside the ED technique for PRCV and the PFU technique for BoHV-1. Two practical applications are detailed: investigating PRCV inactivation on inert surfaces and examining impaired BoHV-1 replication by acyclovir.

## Methodology

**Figure 1.**
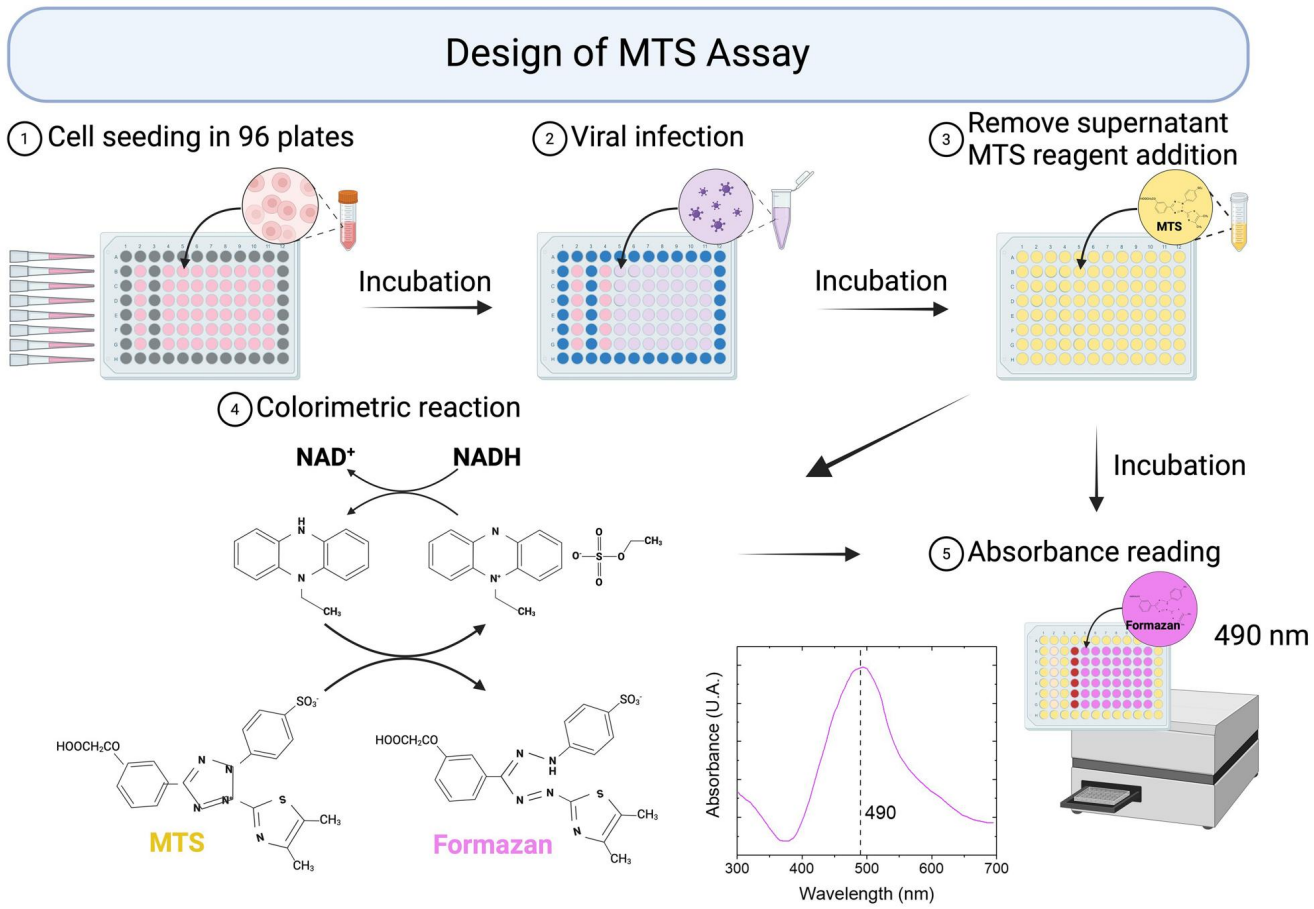
Schematic illustration of the MTS assay and these main steps. (1) Cell seeding in 96-well plate, (2) viral infection, (3) addition of MTS reagent, (4) reduction reaction leading to the production of formazan, and (5) absorbance measurement at a wavelength of 490 nm (see the absorption spectrum).

The MTS assay assesses the metabolic activity of cells and can be applied to evaluate indirectly the virus infectivity within a given sample. Figure 1 illustrates the main steps of the protocol required to carry out the methodology described in this manuscript. The approach relies on the reduction of a tetrazolium salt such MTS reagent to formazan dyes that remain soluble (step 4). This conversion is proportional to NADH concentration within living cells. An intermediate electron acceptor such as phenazine ethyl sulfate is reduced in proportion to the NADH level. The accumulation of the reduced form triggers the MTS reduction into formazan in the culture medium [7, 21]. Since MTS and formazan have different optical properties, a colorimetric quantification can be used to evaluate the chemical reaction yield. Absorbance measurement at a wavelength of 490 nm (the maximal absorption wavelength of formazan) therefore enables formazan quantification, which is proportional to cells metabolic activity. This tetrazolium reduction makes the analysis a colorimetric assay, with the MTS dye being yellow gold, the formazan being brown, and all wavelength values falling between these two colors.

To evaluate the viral load of viruses that induce cell death upon replication, one can determine the survival rate of a fixed number of cells incubated with a viral suspension to estimate the quantity of infectious viruses. The protocol is built around five main stages (Fig. 1). (i) A fixed number of cells is seeded in a 96-well plate, distributed evenly within each well. A post-seeding incubation serves to minimize cellular responses to stress, maintain their viability, and create a standardized environment to optimize the infection. (ii) Cells are exposed to the virus-containing solutions(s) whose titers are to be determined. Exposed cells are further incubated over the time required to trigger the virus induced infectivity. This timing depends on the viral species and the target cells and may have to be determined empirically. The evaluation of this viral infection by MTS reading includes the 3 next steps. (iii) For better homogeneity during the addition of MTS reagent in each well, it is recommended to empty the wells and add a homogeneous mix containing MTS solution and culture medium. This allows, on the one hand, a balanced distribution of the MTS within the well, but also eliminates the dead cells which have detached from the plate, and which are removed with the supernatant. (iv) In metabolically active cells, the reduction of MTS produces formazan. (v) In the final step, absorbance measurement at 490 nm provides information on the metabolic activity of cells post-infection. This value is proportional to the number of remaining living cells, and this is inversely proportional to the number of infectious viruses added in step 2. Using a previously established cell viability-viral dose calibration, it is possible to determine the viral titer.

After measurement, absorbance is measured for each condition. To evaluate the cell viability, i.e. the percentage of viable cells, suitable controls are considered in the following formula:

(1) $$\mathrm{Cell}\;\mathrm{viability}\;(\%)=\;\frac{A_{\mathrm{tested}}-A_{\mathrm{MTS}}}{A_{\mathrm{CTRL}100\%}-A_{\mathrm{MTS}}}\times 100$$

where the background ($A_{\mathrm{MTS}})$ corresponds only to the measurement of MTS-medium solution without cells and viruses in the well. The positive control ($A_{\mathrm{CTRL}0\%})$ indicates a complete virus toxicity toward cells. This value is determined via wells containing cells infected with an excess viral dose or brought into contact with a harmful chemical compound. The negative control ($A_{\mathrm{CTRL}100\%})$ means a total viability with an absorbance value of the mock-infected cells. All absorbance measurements must be between the negative and positive control values. Annex 1 in supplementary information’s details these different concepts.

Six main factors modulate the biological response and need to be optimized in the assay design: (i) the cellular concentration to be seeded per well, (ii) the cell-growth time between seeding and infection, (iii) the viral load that infects these cells, (iv) the post-infection time required for the viruses to cause CPE on these cells, and finally, (v) the assay-time required for the conversion of MTS to formazan, and (vi) the amount of MTS solution brought into contact with the cells. These various parameters depend on both the type of virus and the cells used, as well as their quantities [22]. The Ishikawa diagram in Figure S1 in the Supplementary information presents, under an approach of causes-and-effect, these parameters as well as other factors that may influence results to design a robust HTS assay which evaluates viral infectivity. A plate plan is also proposed in the appendix, in Figure S2 in the Supplementary information.

## Case study 

Viral titration of PRCV and BoHV using the HTS assay was conducted with ST cells and MDBK cells, respectively. The gold standard methods, ED and PFU assays, were performed alongside the MTS technique to compare and validate this high-throughput assay for these two viral species and cell types.

### Materials and methods

#### Cells and virus

ST cells (ATCC CRL-1746, RRID: CVCL_2204) and MDBK cells (ATCC CCL-22, RRID: CVCL_0421) were maintained at 37°C and 5% O_2_ in Eagle’s Minimum Essential Medium (EMEM) supplemented with 10% (50 ml/500 ml) Fetal Bovine Serum (FBS), l-glutamine (292 mg/L), Penicillin (100 U/ml), Streptomycin (100 µg/mL) but also 1 mM sodium pyruvate (only for ST cells) and 1 mM Non-Essential Amino Acid (NEAA; only for MDBK cells). The viruses are the Porcine Respiratory Coronavirus virus (Belgian PRCV -91V44) and the Bovine alpha Herpesvirus 1 (Cooper strain obtained from the University of Liège; Pr. Laurent Gillet, Immunology-Vaccinology department). The PRCV viral working stock was produced in ST cells during an incubation period of 5 days while BoHV-1 stock in MDBK cells, for 2 days. The medium used for infection is Eagle’s Minimum Essential Medium (EMEM) supplemented with 1% (5 ml/500 ml) Fetal Bovine Serum (FBS), l-glutamine (292 mg/L), Penicillin (100 U/ml), Streptomycin (100 µg/mL) but also 1 mM sodium pyruvate (only for ST cells) and 1 mM Non-Essential Amino Acid (NEAA; only for MDBK cells). The supernatant was harvested and clarified by centrifugation (300 g for PRCV and 1000 G for BoHV-1) at 4°C, the viral stock was aliquoted and stored at −80°C until further processing. The virus stock titers were evaluated at $5.5\times 10^{5}\;$TCID_50_/ml (PRCV) and $1.98\times 10^{8}$ PFU/ml (BoHV-1).

### MTS assay

Prior to infection, cells were seeded at density of 6000 (ST cells) or 10 000 (MDBK cells) per well of 96-well-plate using maintenance medium as previously described. After 24 hours, the medium was removed, and each well was infected with a corresponding viral inoculum diluted with EMEM containing 1% FBS (5/500 ml). The concentrations used were 50, 100, 200, 1000, 1500, 2000, 3000, and 5000 TCID_50_/ml in a final volume of 100 µl for PRCV, or 50, 100, 500, 1000, 5000, 10 000, 40 000 PFU/ml in a final volume of 50 µl for BoHV-1. The plates were then incubated at 37°C with 5% CO_2_. After two hours, culture medium containing 5% FBS (25 ml/500 ml) was added to each well. Three days for PRCV and two days for BoHV-1 post-infection, cell viability was measured using a CellTiter-96^®^ Aqueous cell proliferation assay kit (Promega, Madison WI) according to the manufacturer instructions. The media was removed from each well before adding a mix solution (1:6) of the MTS solution and EMEM media in a final volume of 120 µl, which was then incubated for 1.5 h. Absorbance was measured at a wavelength of 490 nm using a SpectraMax iD3 Microplate Reader (Molecular Devices, San Jose, CA, USA, RRID: SCR_023920). The cell viability was determined by the formula (1) presented above. The viral load curve was determined in three independent experiments with three replicates per viral dose in each experiment. The positive control $\left(A_{\mathrm{CTRL}0\%}\right)$ was treated with the detergent Triton X-100 for 15 min at ambient temperature, leading to complete cell lysis.

### Endpoint dilution assay (ED)

ST cells were seeded in 96-well plates with the culture medium at 37°C and 5% CO_2_, 48 h before infection. The PRCV viral inoculum at range between 50 and 5000 TCID_50_/ml were submitted to 10-fold serial dilutions ($10^{0}-10^{-7}$). These eight dilutions were added to semiconfluent cell monolayers for the next five days (six replicates per dilution). These infections were carried out with EMEM media containing 1% (5/500 ml) FBS (100 µl) for two hours, and EMEM containing 5% FBS (150 µl) was added to favor cell growth. Five days post-infection, the CPE was evaluated using an optical microscope, and the viral titers were calculated using the Reed-Muench method [2, 3], expressed as TCID_50_/ml. The limit of detection corresponds to 20 TCID_50_/ml.

### PFU assay

The viral titration by PFU assay was carried out using MDBK cells and BoHV-1, obtained from the University of Liège (Pr Laurent Gillet, Immunology-Vaccinology department). Cells were cultured in EMEM media supplemented with 10% FBS (50/500 ml) at 37°C in a humidified atmosphere containing 5% CO_2_. Tenfold serial dilutions of the virus stock were prepared in infection media, and 200 µl of each dilution was inoculated onto confluent cell monolayers in 24-well plates. Following an incubation period of 2 hours at 37°C, the cells were overlaid with culture medium (1 ml) containing MEM, 10% FBS, and 0.8% Carboxymethyl-Cellulose Low Viscosity (CMC). Plates were incubated for 72 hours under identical conditions. Subsequently, the cells were fixed with 4% paraformaldehyde and stained with crystal violet. Plaques were enumerated, and the viral titer was calculated as PFU/ml using the following formula:

(2) $$\mathrm{PFU}/\mathrm{ml}=\frac{\mathrm{Number}\;\mathrm{of}\;\mathrm{plaques}}{\mathrm{Dilution}\;\mathrm{factor}\;\times \mathrm{Volume}\;\mathrm{inoculated}\;(\mathrm{ml})}$$

All experiments were performed in triplicate to ensure reproducibility and accuracy.

### Survival of PRCV on inert surfaces

The persistence of PRCV virus on inert surfaces was examined using stainless steel (304), polymethyl methacrylate acrylic (acrylic glass), and aluminum alloy (AW-6082). To reflect the viral load found in fomites replicate real-world conditions, 20 μl of infection media with 2000 TCID50/ml (the upper limit of detection for PRCV) was applied onto each surface, forming five 4 μl droplets to maximize surface–liquid contact [20]. The temperature and relative humidity were maintained at 22°C and 50%, respectively. Samples were collected at 0, 2, 4, 6, 8, 12, and 24 hours post viral deposition by immersing each surface in 1 mL of EMEM and storing them at 4°C for subsequent virus titration using an MTS-based assay. Additionally, a parallel experiment was conducted using an ED assay to titer virus inoculated at 8000 TCID_50_/ml. The ED assay was conducted in triplicate, while the MTS-based assay was conducted once.

The time needed to achieve a 90% reduction in inoculated titer (D90, analogous to the D-value) for each material was determined using the Weibull model [23]. This model provides a more comprehensive analysis of viral inactivation kinetics than the first-order kinetic model [24], which assumes a constant inactivation rate. The Weibull model, a two-parameter non-linear model, is described by the equation:

(3) $${\mathrm{Log}}_{10}\left(\frac{N(t)}{N_{0}}\right)=\frac{-1}{2.303}{\left(\frac{t}{a}\right)}^{b}$$

where *N*(*t*) is the viral concentration (TCID_50_/ml) at time *t*, *N*_0_ is the initial inoculum titer (TCID_50_/ml), *a* is the scale parameter influencing the position of the survival curve along the time axis, and *b* is the shape parameter determining the curvature of the survival curve. A value of *b* = 1 corresponds to exponential decay, values of *b* < 1 to faster initial decay, and values of *b* > 1 to slower initial decay. The D_90_ values were calculated from the fitted Weibull model parameters using the following formula (Van Boekel, 2002):

(4) $$D_{90}=a{\left(2.303\right)}^{\left(\frac{1}{b}\right)}.$$

### Evaluation of the antiviral effect of acyclovir impairing BoHV-1

MDBK cells were seeded at a density of 10 000 cells per well in a 96-well plate and incubated for 24 hours at 37°C with 5% (v/v) CO_2_ to allow adherence. The cells were cultured in EMEM supplemented with 10% FBS (50/500 ml). Subsequently, the cells were infected with BoHV-1 at a multiplicity of infection (MOI) of 0.5. Acyclovir, prepared in EMEM containing 10% FBS, was added 2 hours post-infection at final concentrations of 0, 50, 100, and 200 µM. Following the addition of acyclovir, cells were incubated for an additional 24 hours under the same conditions. Viral replication and cell viability were evaluated using the MTS assay for only one experiment 5 technical replicates.

To facilitate reproduction of this work, the complete protocols for PRCV and BoHV-1 are provided for the reader in Supplementary information (section: Optimization of cell and viral cultures for PRCV and BoHV-1, and, Figure S2 in the Supplementary information) with a list of all reagents used, along with their sources and product numbers (Table S2 in the Supplementary information).

### Data analysis

The results were obtained, experimentally, from three replicates for the TCID_50_ method and three different plates for the MTS method. Each experiment was independently repeated three times. The statistical analysis was performed using a two-way ANOVA. The performance measures in HTS assays are evaluated by statistical parameters such as Z′ factor, signal-to-noise ratio (S/N), the signal-to-background ratio (S/B) and the relative standard deviation (RSD) [26–28]:

(5) $$Z^{\prime }=1-\frac{3({SD}_{\mathrm{CTRL}0\%}+{SD}_{\mathrm{CTRL}100\%})}{A_{\mathrm{CTRL}0\%}-A_{\mathrm{CRTL}100\%}}$$

 

(6) S/N=ACTRL100%-ACTRL0%(SDCTRL100%2-SDCTRL0%2)12

 

(7) $$S/B=\frac{A_{\mathrm{CTRL}100\%}}{A_{\mathrm{CTRL}0\%}}$$

where SD is the standard deviation, A is the average, and CRTL0% and CTRL100% are the positive and negative controls, respectively. If Z′ is > 0.5, the test is deemed excellent, 0 < Z′ < 0.5 the test is feasible, Z′ = 0 the test is a failure with two binary responses, or Z′ < 0, the test is inadequate [26]. For the other parameters, it is recommended to have S/B > 3, S/N > 10. [29].

### Results

To accurately determine the viral titer of a viral solution by quantifying host cell metabolic activity using the MTS assay, it is crucial to optimize a calibration curve that correlates a known viral titer with the corresponding absorbance measurements. The following six critical parameters must be considered during this optimization process:

- the cell concentration to be seeded per well,
- the duration of cell growth between seeding and infection,
- the viral load used to infect the cells,
- the post-infection incubation time required for the virus to induce CPEs in the cells,
- the assay duration required for the conversion of MTS to formazan,
- the amount of MTS solution brought into contact with the cells.

**Figure 2.**
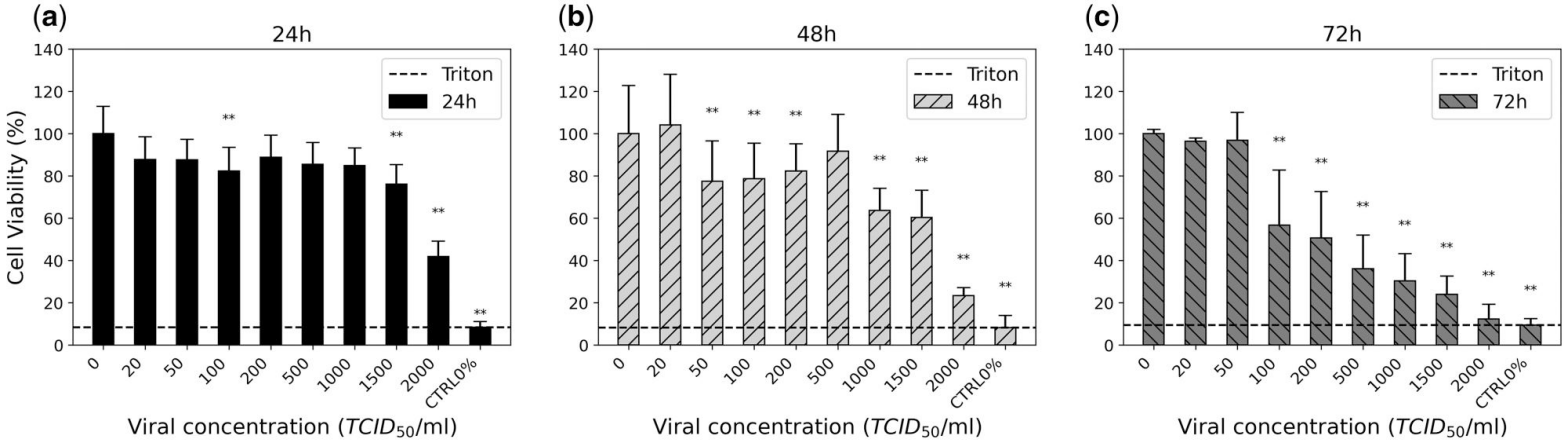
Optimization of the MTS assay. Investigation of post-infection timing: cell viability as a percentage at different post-infection time points for viral concentrations ranging from 0 to 2000 ${\mathrm{TCID}}_{50}/\mathrm{ml}$ after (**a**) 24 h, (**b**) 48 h, and (**c**) 72 h. The infectivity between different viral doses alone was highly significant above the concentration of 1500 TCID_50_/ml for 24 h, 1000 TCID_50_/ml for 48 h, and 50 TCID_50_/ml for 72 h. Differences of mean absorbance values between viral concentrations were highly significant: *P* < .01 for ANOVA. Errors bars indicate the SD of 5 cell viability values analyzed simultaneously. The experiment was performed once. The following symbols were used for the comparison between the negative control (0 TCID_50_/ml) and different doses: **P ≤ .01, non-statistically significant results were not indicated.

This optimization process is illustrated in Fig. 2 for the PRCV viral species, where two parameters are kept constant: a post-seeding incubation time of 24 hours and a fixed cell density of 6000 cells per well. A similar procedure was carried out for BoHV-1 (data not shown) with a similar post seeding time and a cell density of 10 000 cells per well.

Figure 2 shows the cell viability relative to Mock-treated cells after different post-infection (pi) times and for different viral concentrations ranging from 0 to 2000 TCID_50_/ml. It is observable that at 24hpi, between 0 and 1500 TCID_50_/ml, the cell viability is ∼85%–90%, forming a plateau. At the highest dose, viability falls to 40%. At 48hpi, the influence of the virus on cells is detected for viral inoculum equal or superior to 1000 TCID_50_. This plateau is reduced. Beyond 1500 TCID_50_/ml, the cell viability is close to the value of the positive control (10%), where the cells have been killed with a detergent. These values are very different from low-dose virus values: it is therefore possible to discriminate between low and high doses. At 72 hpi, a significant impact on the cell viability according to the viral dose was observed on values ranging from 50 to 2000 TCID_50_/ml, with cell viability progressively dropping from 98% at 20 TCID_50_/ml to 10% at 2000 TCID_50_/ml. Only values below 50 TCID_50_/ml still show important cell viability of 90%. Microscopic observations confirmed these results (Figure S3 in the Supplementary information).

**Figure 3.**
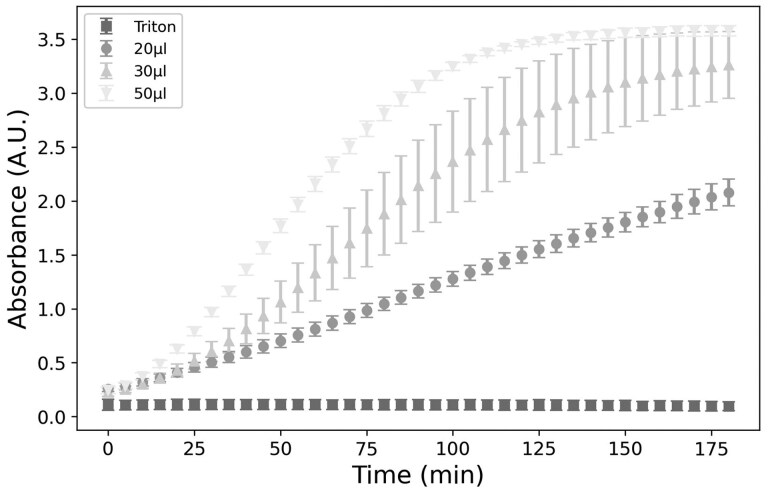
Examination of the time after MTS addition: absorbance of the negative control (monolayer cells without viral infection) using different MTS solutions (20, 30, and 50 µl) and the positive control (monolayer cells without viral infection but with detergent). In all cases, the initial cell concentration is 6000 cells in each well. Errors bars indicate the SD of 8 absorbance values analyzed simultaneously. The experiment was performed once.

The quantity and incubation time required for the MTS reagent to convert to formazan are illustrated on Fig. 3. Three volumes were investigated: 20, 30, and 50 µl of MTS solution for a reading at 72 hours post-infection for the positive control (cells without virus). For a fixed incubation time, an increase in the reagent volume was associated with an increase in the absorbance. It was also observed that, after a certain time, depending on the concentration of MTS, a plateau was reached, indicating a saturation. The latter occurs more rapidly as the quantity of the solution increases. This plateau may be due to either the limitations of a detector and the properties of the spectrophotometer, or, the total conversion of MTS into formazan. Consequently, to optimize this assay, it was necessary to define the incubation time while remaining within the linear regime.

The viral-load response was investigated, and the viral-load curve was determined for the derived experimental MTS parameters. These were as follows, listed respectively for PRCV and BoHV-1 respectively: (i) 6000 and 10 000 seeded for each well, (ii) absorbance readings at 72 and 48 hours pi, (iii) viral concentrations ranging from 0 to 2000 TCID50/ml and 500 to 10^6^ PFU/ml. For both viruses, (iv) the post-seeding incubation time (24 hours), (v) the MTS volume (20 µl), and (vi) the MTS incubation time (90 minutes) were consistent. For comparison, the conventional methods employed were ED technique for PRCV and PFU assay for BoHV-1.

**Figure 4.**
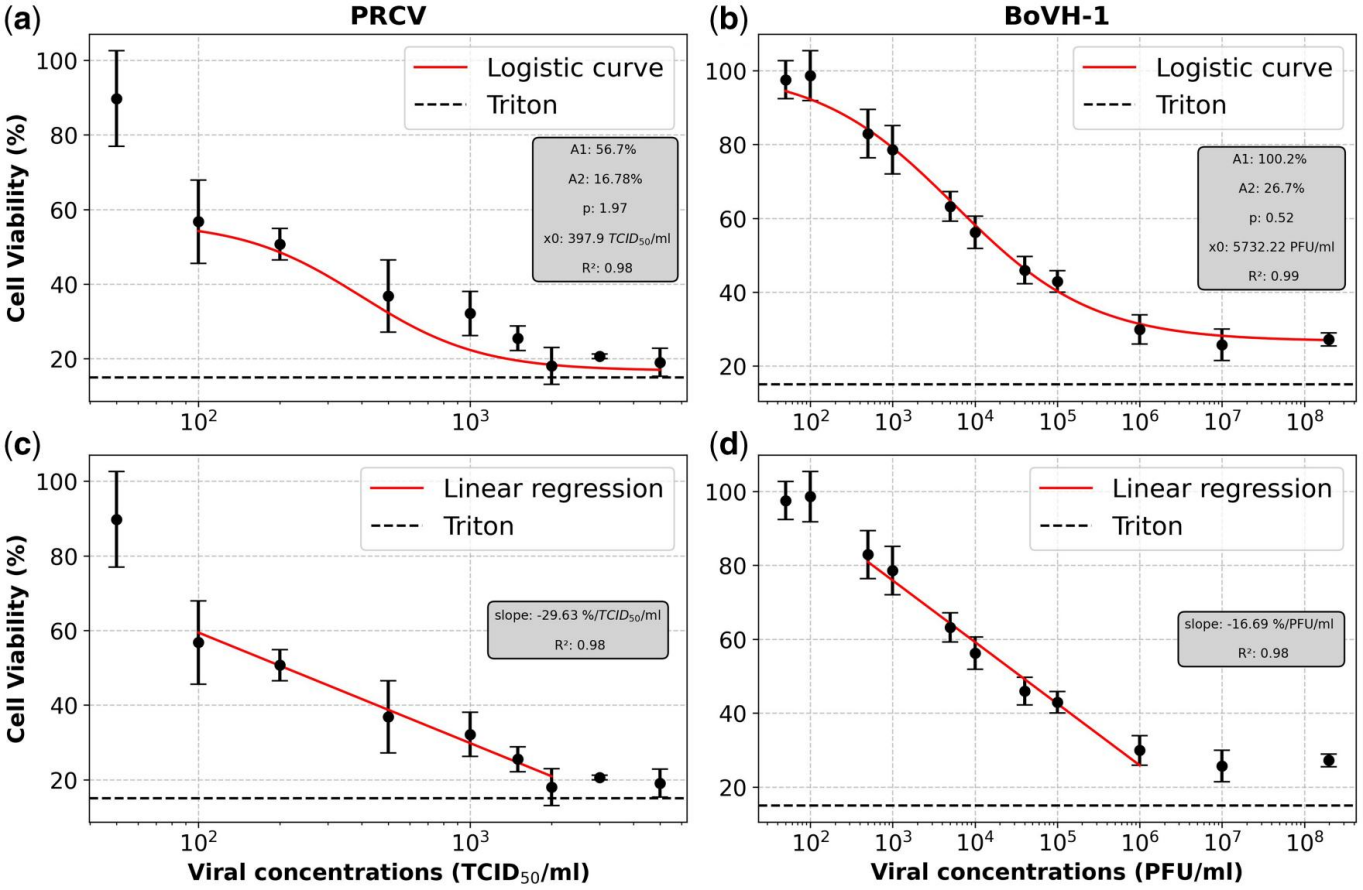
Cell viability curve as a function of virus load for PRCV (**a**, **c**) and for BoHV-1 (**b**, **d**). The horizontal dashed black line represents the value of positive control, which consists of cells treated with chemical compound (triton). Each value corresponds to the mean of five independent assays performed simultaneously. Errors bars indicate the SD for 3 replicates. The linear regressions for these two graphs reveal significant correlations, with Pearson coefficients of 0.99 (*P* < .05, *R*^2^ = 0.98) for both.

Figure 4a and b show cell viability against viral concentration for PRCV (TCID50/ml) and BoHV-1 (PFU/ml). The dashed black line marks the positive control threshold: 15% for PRCV and 10% for BoHV-1. At low viral concentrations (below 50 TCID50/mL for PRCV or 500 PFU/mL for BoHV-1), cell viability remains stable. As viral concentration rises, cell viability drops to 16% at 2000 TCID50/ml for PRCV and 26% at 10^6^ PFU/ml for BoHV-1, nearing the threshold. Higher doses approach the positive control thresholds, with a linear relationship between 100 and 2000 TCID50/ml for PRCV and 500 and 10^6^ PFU/ml for BoHV-1 (Fig. 3c and d). Linear regressions show strong correlations with Pearson coefficients of 0.99 (*P* < .05, *R*^2^ = 0.98), for both viruses.

**Figure 5.**
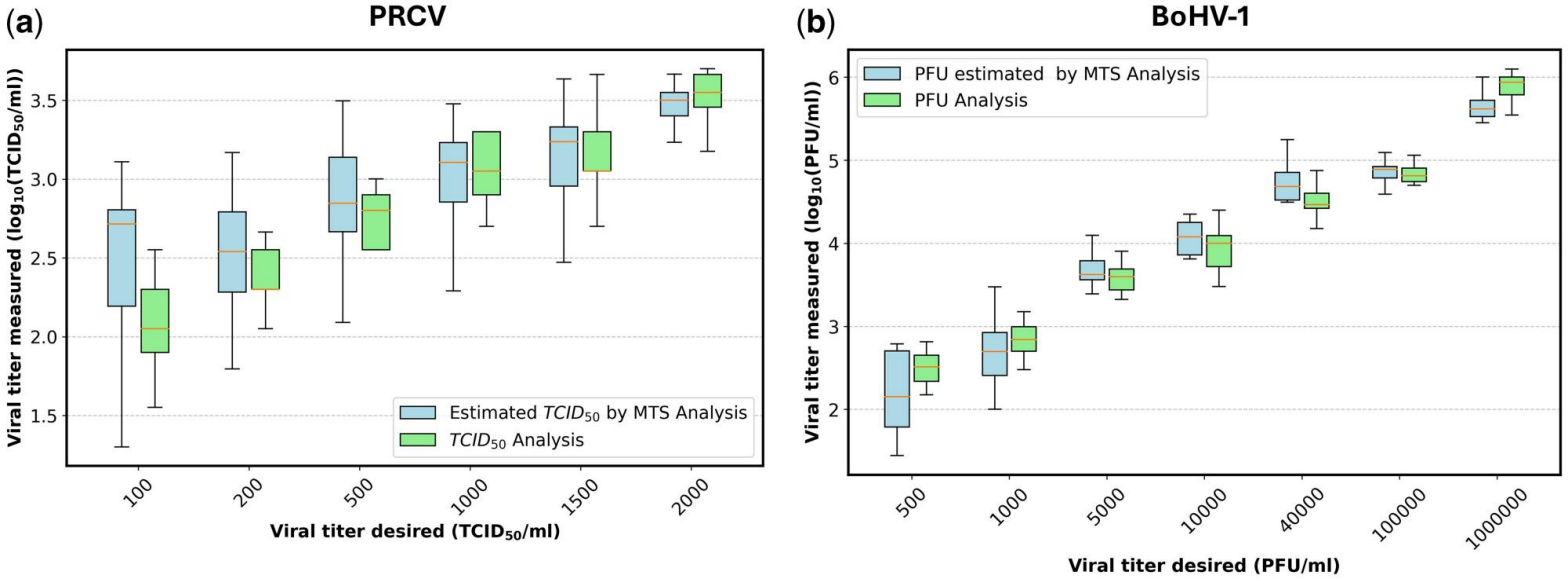
Comparison of viral titers estimated by tetrazolium assay and endpoint dilution method for PRCV (**a**), or by plaque forming unit method for BoHV-1 (**b**). ANOVA-2 results: the compared techniques show no significant difference (*P* = .68 for PRCV, *P* = .19 for BoHV-1), nor does their interaction with the desired PFU (*P* = .72, *P* = .26). Each value corresponds to the mean of five independent assays performed simultaneously. Errors bars indicate the SD for 6 replicates for PRCV and 3 replicates for BoHV-1. In contrast, the viral theorical titer has a highly significant effect (*P* < .001).

To validate the optimized MTS assay, we compared it with conventional methods. Figure 5 shows the results of MTS analysis (blue) and two reference methods (green): ED assay for PRCV and PFU assay for BoHV-1. For these viruses and on the linear regime described in Fig. 3, the two-factor ANOVA analysis revealed no significant difference between the techniques (*P* = .68 and .19) and no significant interaction between the technique and the viral titer desired (*P* = .72 and .26), but it showed a significant effect of the viral titer desired (*P* < .001). This demonstrates the similarity between the three methods for titrating the same viral solution. However, HTS tends to overestimate titers compared to the other two methods.

To validate the robustness of our assay, the factors, Z′, S/N, and S/B were determined. Compared with the threshold values, it indicates that MTS assay is excellent in the case of PRCV. For BoHV-1, the feasibility is strengthened by the excellent signal-to-noise and the signal-to-background ratios.

**Figure 6.**
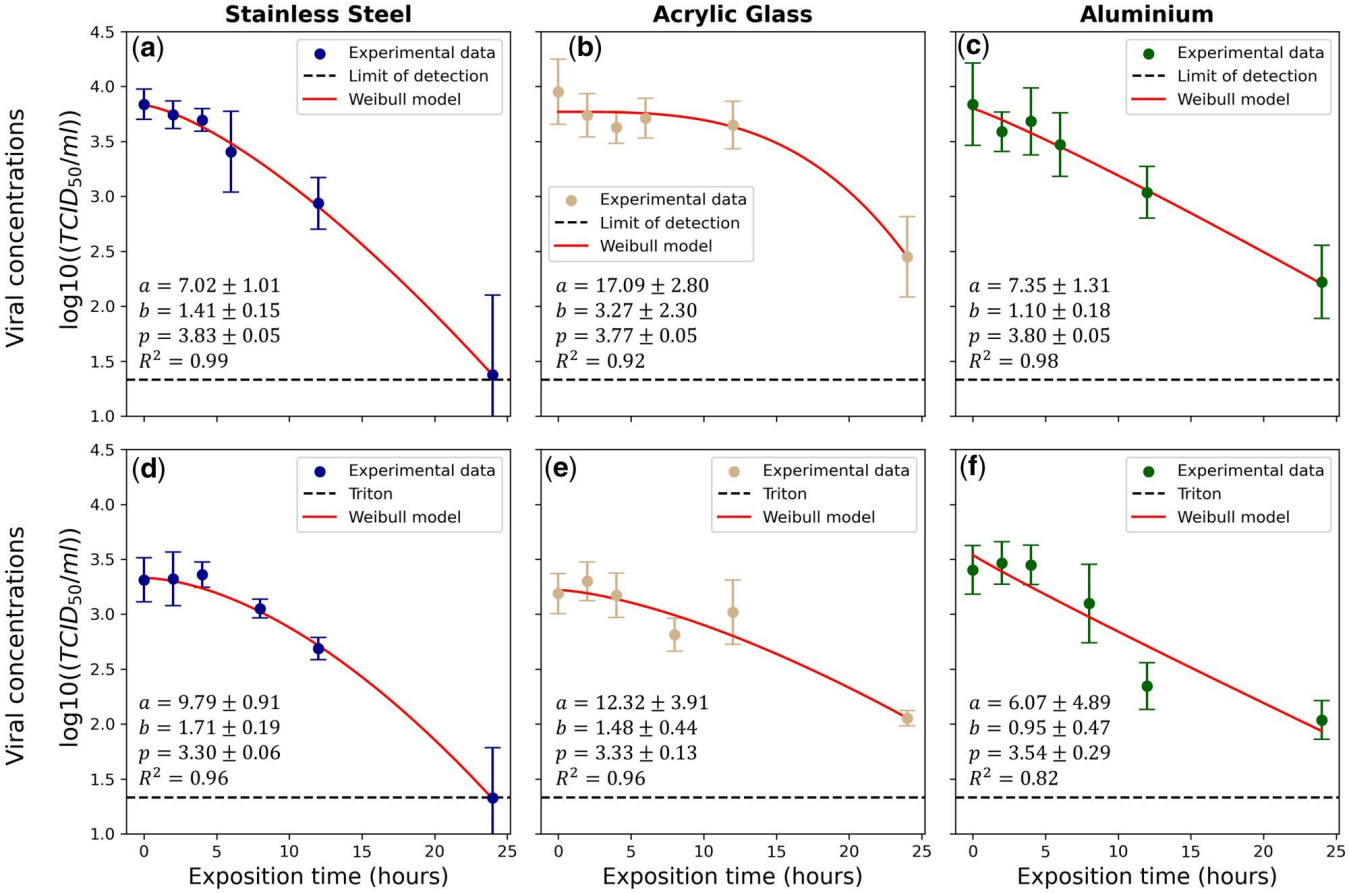
Potential application: study of the survival of viruses on inert surfaces, (**a**, **d**) stainless steel, (**b**, **e**) acrylic glass, and (**c**, **f**) aluminum alloy The results are obtained by ED titration (a–c) and MTS (d–f). The ED assay was conducted in triplicate, while the MTS-based assay was conducted once.

This HTS assay can be used to determine the titer of viral solutions collected from surfaces to evaluate virus inactivation kinetics. Figure 6 shows results on stainless steel (a, d), acrylic glass (b, e), and aluminium alloy (c, f) after titration by the ED method (a–c) and the MTS-based assay (d–f). For the MTS assay, cell viability from absorbance readings was converted to estimated viral load using the calibration curve from Fig. 4. Survival curves showed similar trends for both techniques. From 0 to 5 h, doses remained constant until 5 h for stainless steel and aluminium, after, these surfaces showed a specific decrease. Acrylic glass showed a plateau up to 12 hours. D_90_ values using ED and HTS assays were 12.68±1.98 h and 15.94±1.72 h for stainless steel, 22.05±5.35 h and 21.64±7.77 h for acrylic glass, and 15.68±3.42 h and 14.60±13.33 h for aluminium. The relatively high uncertainties in the obtained values are due to the fact that only a single experiment was conducted, compared to three for the reference method. Nevertheless, based on the shape of the curves, there are strong similarities, highlighting the potential of HTS for quantifying virus inactivation on surfaces.

**Figure 7.**
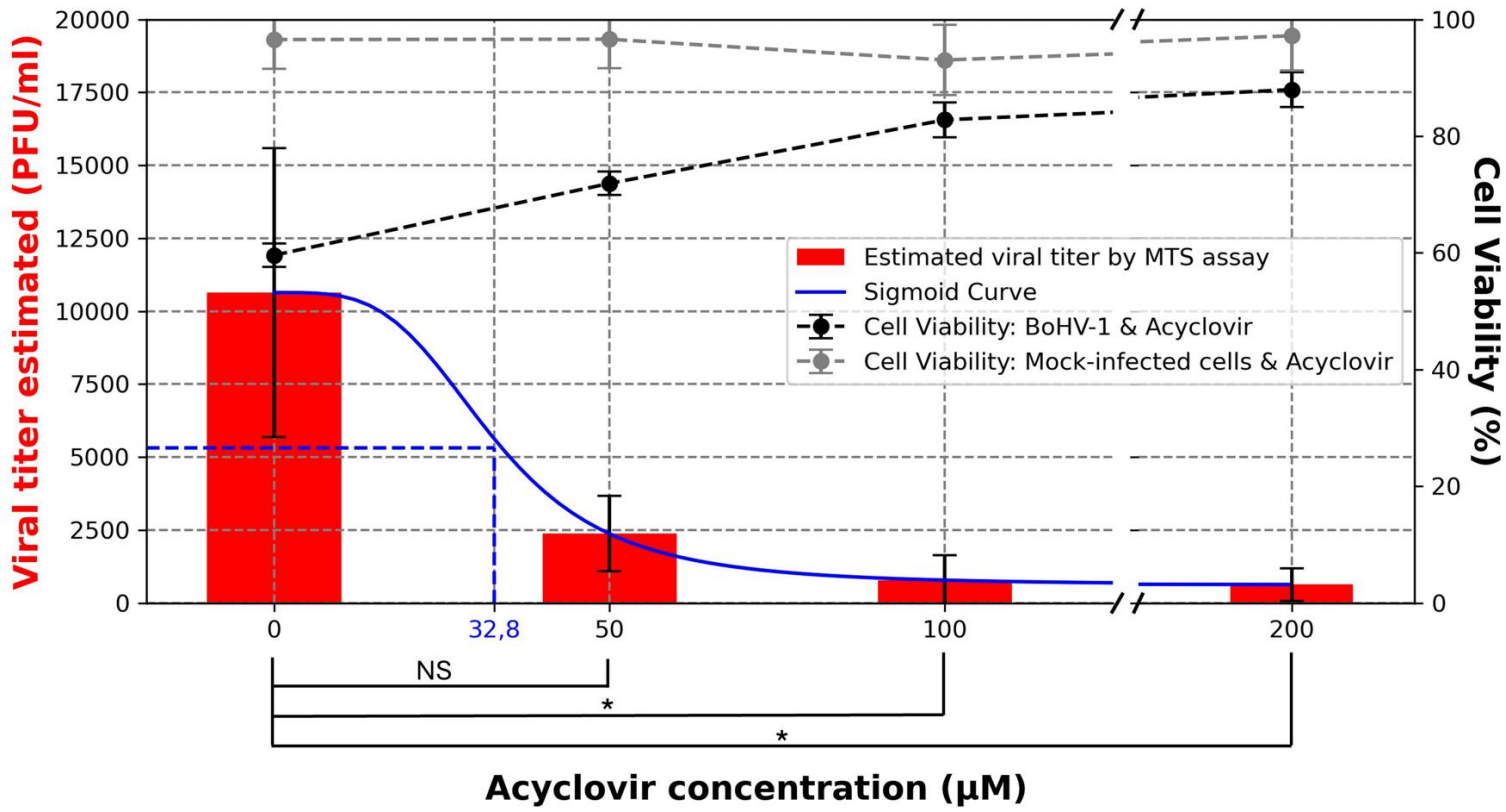
Study of viral inactivation of BoHV-1 virus as a function of acyclovir concentration. The left *y*-axis shows the estimated viral titer (PFU/ml) determined by MTS after 24 hours in acyclovir (red). The right *y*-axis displays cell viability for infected (black) and mock-infected cells (grey). The left *y*-axis shows the number of PFU/ml as estimated by the HTS assay after 24 hours of incubation with various acyclovir concentrations. The right *y*-axis displays cell viability, measured by metabolic activity, for both infected and mock-infected cells. Viral replication and cell viability were evaluated for only one experiment by 5 technical replicates. NS: not statistically significant; *P≤.05.

Another potential application of this HTS assay is drug screening in pharmaceutical field. Acyclovir, an antiviral used for herpesvirus infections, inhibits viral DNA replication by targeting the thymidine kinase and the viral DNA polymerase. Figure 7 presents a study examining the effect of acyclovir on Bovine alpha Herpesvirus 1 inactivation kinetics using MDBK cells. The left y-axis shows the number of PFU/ml as estimated by the HTS assay after 24 hours of incubation with various acyclovir concentrations. The right y-axis displays cell viability, measured by metabolic activity, for both infected and mock-infected cells. Higher acyclovir concentrations resulted in increased cell viability and decreased viral load, showing a dose-dependent effect. The effective concentration (EC50) values were determined to a sigmoid curve and is evaluated at 32.8 µM of acyclovir. The viability of mock-infected MDBK cells remained constant across all tested acyclovir concentrations.

In these two applications, the HTS assay demonstrated a significant time advantage over traditional methods. Specifically, the viral infection step (step 2, Fig. 1) was reduced to less than 1 hour for 80 titrations, thus eliminating the need for laborious serial dilutions required in the ED titration. Overall, the entire assay, including the acyclovir treatment, was completed one day faster than the conventional PFU method, while still maintaining a wide dynamic range of 500 to $10^{6}$ PFU/ml.

### Discussion

The determination of viral titer is essential for understanding the infectious load and dynamics of viral infection. Standard methods for measuring viral titer are often laborious, time-consuming and do not allow a large number of samples to be screened simultaneously. In this paper, the use of tetrazolium reduction assay, a routine cell biology assay, has been extended to applications in virology. The method evaluates the metabolic activity of infected cells and can be used as an effective HTS assay. In this case study, the concentration ranges have been defined from 100 to 2000 TCID_50_/ml for PRCV while from 500 to $10^{6}$ PFU/ml for BoHV-1. This methodology has been compared to conventional ED method and PFU method.

Results have demonstrated that the MTS assay is infection-dose-dependent after 72 hpi (Figs 2c and 4), highlighting CPEs which increase with the viral dose as illustrated in Figure S3 in the Supplementary information [30, 31]. Following these infections, morphological changes in the cell monolayer result in detachment of cells from the bottom of the plate, cell rounding, cell degeneration, or syncytium formation. These effects are virus- and cell-dependent and increase as the incubation time for infection increases. Based on the observations made by [32], ST cells infected with Transmissible Gastro-Enteritis Virus show morphological changes only 20 hpi. From 40 hpi, a disruption of the cell monolayer occurs, leading to a detachment of cells from the plate. This time before reading will depend on the species as well as the quantity of cells and viruses. It will be higher with the number of cells and inversely proportional to virus concentration [33]. For PRCV, 72hpi was determined as the optimal time. Muylkens et *al*. examined the replication kinetics of BoHV-1 in MDBK cells. Their findings indicated that intracellular growth reached a plateau after 20 hours post-infection, suggesting an initial phase of active replication followed by a stable phase. The release of virions into the extracellular medium was exponential and reached a threshold after 35 hours. For practical reasons, this peak timing was considered optimal [34]. Additionally, it was noted that these phenomena vary from strain to strain, as shown by comparing BoHV-1 and BoHV-2. Therefore, a calibration curve must be generated for each viral strain.

The viral load will depend on the type of virus being quantified, the cells used, and their numbers. As illustrated in this study, the analysis range varies significatively between PRCV (100–2000 TCID_50_/ml) and BoHV-1 (500 to $10^{6}$ PFU/ml). Estimates from the literature show that it is possible to have a range of 5–700 TCID_50_ for porcine parvovirus/porcine kidney cells, 5–1200 TCID_50_/ml for canine parvovirus/canine tumor cells, 20–2000 TCID_50_/ml for minute virus of mice/kidney cells [35], as well as a range of 10–1000 TCID_50_/ml for enterovirus/Vero [5]. However, it is possible to obtain larger linear ranges, as demonstrated by Takeuchi et *al.*, who reported a 3-log range for herpes simplex virus in Vero cells, or Muller *et al.*, who reported a range of 200 to 160 000 TCID_50_/ml for Zika virus in Vero cells [31, 36] These works involving MTT (3-[4,5-dimethylthiazol-2-yl]-2,5-diphenyltetrazolium bromide) reagent, showing that it is also possible to optimize this test with other tetrazolium reagents.

The Z′ factor is a value that represents the quality of a high-throughput biological assay by assessing the separation between positive and negative signals. The greater the difference between these values, the greater the Z′ factor (see Fig. 4). In this paper, the factor is estimated at 0.72 for PRCV, characterizing the assay as excellent for PRCV while is evaluated at 0.40 for BoHV-1. This value indicates that the test is acceptable but highlights the need for better separation between the positive and negative controls. One potential approach is to increase the CTRL100% value by extending the incubation time to enhance the transformation of MTS into formazan. Severson *et al*. and Müller *et al*. obtained similar values: 0.68 for SARS-CoV-Vero cells and 0.77 for Zika virus-Vero cells, respectively [31, 37]. Nissen et al. have carried out similar studies but with the WST (Water Soluble Tetrazolium) reagent and have obtained a Z′ value of 0.98 [38].

To validate these methods, it is necessary to make a comparison with conventional titration methods. The results obtained show that the comparison between our HTS assay with MTS solution and the gold-standard method yields a correlation coefficient close to 0.98, either between 100 and 2000 TCID_50_/ml for PRCV and either 500 to $10^{6}$ PFU/ml. Other scientific publications have obtained similar correlations, ranging from 0.94 to 0.99 [5, 35, 39]. The statistical analysis performed, a two-way ANOVA with the technique and the viral titer desired, supports these observations. To the best of the authors’ knowledge, Nissen *et al.* is the only publication that converts cell viability data into TCID50/ml and demonstrates a good correlation for solutions titrated using the tetrazolium WST and ED techniques for Bovine Viral Diarrhea Virus (BVDV), Encephalomyocarditis Virus (EMCV) and Porcine Parvovirus (PPV) [38].

The presented tetrazolium-based assay offers several advantages. Compared to the reference ED assay, the analysis time is reduced from seven to four days for PRCV. The simplicity of the protocol, which does not require dilution or intermediate steps, makes this test user-friendly, allowing for the analysis of a large panel of samples simultaneously, and even enabling process automation [40]. This simpler use also facilitates its implementation in biocontainment laboratories such as BSL-3 and 4 [41]. The ED and PFU assay, based on a subjective visual observation of the CPE, provides an approximate discrete titer, while the MTS assay provides continuous and quantitative data on the infected rate of virus on cells. It is a direct measure of the virus’s infectivity on host cells. Unlike gold-standard method, which requires many manipulations, Heldt *et al*. shows that the user does not impact the experiment and the results obtained [35]. As for Pourianfar *et al*., it shows that using a tetrazolium reagent during reading increases the linear window between the plateaus of 0% and 100% viability of the survival sigmoid [5]. This is because the tetrazolium measurement is more sensitive than subjective observation with the naked eye. Furthermore, unlike HTS systems based on isolated targets such as enzymes or receptors [14], the MTS method is a predictor of cellular absorption and covers the entire virus replication cycle. This test can also be extended to non-adherent cells on the culture plate [36] and bacteriophage viruses [42].

Despite these advantages, this method has some limitations. The MTS test as virus titration method is restricted to viruses and cells that produce CPEs, affecting cell viability [30]. Regarding infectivity, the MTS test may sometimes provide slightly biased information, since it is based on mitochondrial activity, and the stress caused during infection can sometimes be linked to a slight increased activity and may, therefore, be interpreted as an increase in the number of cells [43–45]. Some authors however emphasize that tetrazolium-reduction assays, in general, underestimate the number of living cells when compared to other techniques such as ATP- or DNA-quantification assays [46] The metabolic activity of viable cells may be influenced by different culture conditions, cell types, their growth state, growth-medium composition, and other chemical or biological compounds [47, 48].

The analytical range provided by this assay, ranging from 100 to 2000 TCID_50_/ml or 500 to $10^{6}$ PFU/ml, depends on the viral and cellular species used, as well as various culture conditions and parameters such as incubation time, density of metabolically active cells, or the added MTS concentration. These can be adjusted before expanding or reducing this range to better suit a particular application.

As seen previously, this new technique, compared with reference methods of viral titration, provides non-subjective observations for the viral titer to be determined, enabling a large number of samples to be tested in a short space of time. As demonstrated in this study, potential applications include antiviral surfaces to limit virus spread and drug screening for antiviral compounds. In both applications, the HTS assay significantly outperformed traditional methods in terms of speed. The viral infection step was reduced to less than an hour for 80 titrations, eliminating the need for tedious serial dilutions required in ED and PFU titration. For acyclovir treatment, the entire assay was realized a full day faster than the gold-reference PFU method while still maintaining a large range of 500 to 10^6^ PFU/ml.

For the first application, spreading of viral infections are challenging and the inanimate objects, called fomites, might serve as the potential intermediaries for indirect transmission of viral infections [49–51]. Since the COVID-19 pandemic, many articles had studied the inactivation of the coronavirus SARS-CoV-2 on surfaces under ambient conditions of temperature and relative humidity, 25°C and 50% RH, showing the potential role of these surfaces in viral transmission. Respectively, for stainless steel and acrylic coupons, Van Doremalen et al. obtained estimated D90 values of 18.6 and 22.6 hours while Bonil et al reported a D90 value at 11.09 hours for stainless steel and a comparable result for acrylic glass with 21.6 hours [20, 52]. Pottage et al. had obtained 17.8 and 16.8 hours for two variants of SARS-CoV-2 on stainless steel [53]. Hassan et al. showed that with an inoculum of 1000 TCID_50_, no viruses were detectable on a nanostructured aluminum surface, whereas on a flat aluminum surface, the virus remained viable for 48 hours [54]. Sizun *et al.* reported a survival time of 12 hours for HuCoV-229E on aluminum [55]. D values were observed in the same range by applying HTS titration. Contrasting with studies based on time consuming titration assays, HTS can be an effective method for rapidly testing a large number of surfaces to better understand the inactivation time of viral species on inert surfaces or to optimize antimicrobial surfaces for combating pandemics and hospital-acquired infections in healthcare settings [56, 57].

In the second context, HTS were applied to study the effect of antiviral compounds against the BoHV-1, using MDBK cells. The cytotoxic concentration 50% (CC50) of acyclovir against these cells was established at 989 µM, considerably higher than the concentrations used in this study [58]. Median effective concentration (EC50) determined from our assay is 32.8 µM against BoHV-1 at 48 h pi using a MOI of 0.5. By comparison, Yuan *et al.* showed that acyclovir at a concentration of 10 µM had no inhibitory effect against BoHV-1 at an MOI of 1 [59]. However, they demonstrated a synergistic effect when combined with intracellular non-enzymatic antioxidant glutathione (GSH) at 10 mM, with increasing concentrations of acyclovir enhancing the effect. The median effective concentration (EC50) was determined, by Pinto *et al.*, at 166 µM in a model involving MDBK cells and BoHV-5 without indicating the MOI. Using feline corneal explant model, ED50 was assessed at 78.6 µM against another alpha herpesvirus the feline herpesvirus type-1 (FHV-1). In this assay, the number of cells exposed to viral infection was not determined, impairing the possibility to assess the MOI [58, 60]. Altogether, this reveals that the available literature is scarce, and the varying models used across studies prevent a consensus from emerging to advance research. This challenge stems from the lack of standardized techniques and analytical processes. HTS can address this gap by providing standardization while offering key advantages: ease of use, high automation potential, the ability to test a large number of samples for better result comparison, and a significant time advantage over conventional titration methods.

## Conclusion

In conclusion, a new approach for titrating a wide range of viral solutions is proposed, based on a tetrazolium-reduction assay. An alternative to conventional tests that are laborious, subjective, time-consuming, and require significant resources and consumables, this test is being developed as a HTS assay that is easier to use and allows a large number of samples to be assessed. Two case studies of the assay were conducted: one with porcine PRCV virus in ST cells and another with bovine BoHV-1 virus in MDBK cells. Forward analysis windows were established, respectively, from 100 to 2000 TCID50/ml and from 500 to $10^{6}$ PFU/ml. Correlation analysis between these new titration methods and the conventional ED and PFU techniques demonstrated strong agreement in the results. Two potential applications of this high-throughput assay include screening for antiviral drugs and investigating viral survival on inert materials.

## Supplementary Material

bpaf049_Supplementary_Data

## Data Availability

Data not publicly available.
