# Supplementary material for: Development of high-throughput screening viral titration assay: Proof of concept through two surrogate viruses of human pathogens
Source: Biol Methods Protoc. 2025 Jun 17;10(1):bpaf049. doi: 10.1093/biomethods/bpaf049 (PMC12417078; doi:10.1093/biomethods/bpaf049)
Supplement: bpaf049_Supplementary_Data [file bpaf049_supplementary_data.docx]

Table S1: Comparison of various traditional methods for determining viral titers and the high-throughput screening assay utilizing the MTS reagent.

|  | **This work** | **Conventional methods** | | | | | | | | |
| --- | --- | --- | --- | --- | --- | --- | --- | --- | --- | --- |
|  |  |  | | | | | | | | |
|  |  | Group 1  Cell-based assays for measuring viral infectivity | | | Group 2  Gene or protein expression assays to detect virus presence | | | | Group 3  Direct counting of viral particles | |
| **Method:** | **Tetrazolium assay** | **TDIC50** | **Plaque Assay (PFU)** | **Immunofluorescence foci assay** | **qPCR** | **ELISA** | **Hemagglutination assay** | **Immunofluorescence** | **Viral flow cytometry** | **Electron microscopy** |
| Measure | Metabolic activities of cells | CPE | CPE | CPE | Genome relative | Specific viral proteins | Agglutination of red blood cells by viruses | Specific viral proteins by fluorescent labeling | Count of active and defective viruses | Observation of individual particles (active and defective) |
| Duration | 3-7 days | 5-12 days | 5-12 days | 5-9 days | 1 day | 1 day | 1 day | 1 day | 1 day | 1 day |
| Sensitivity | High | High | High | High | High | Variable | Variable | Variable | Limited | Limited |
| Specific equipment | Spectrophotometer and tetrazolium kit | Optical microscope, use of a lot of consumables | Use of a lot of consumables | Fluorescent antibodies, use of a lot of consumables | qPCR and virus-specific primers; viral standards to quantification | Spectrophotometer, ELISA reagents (Commercial kits) | Microplates, optical microscope | Fluorescence microscope | Specific flow Cytometer and specific markers | Electron microscope |
| Costs* | Low | Medium | Medium | High | Low | Medium | Low | High | High | Medium |
| Human Labor | Low | High | High | High | Low | Low | Low | Medium | Low | Low |
| Reproductibility | Medium | Medium | Medium | Medium | High | High | Medium | Poor | High | Variable |
| Variability | Low | Medium | Medium | Medium | Low | Low | Good | Medium | Low | Variable |
| Quantity of samples | High | Limited | Limited | Limited | High | Medium | Medium | Medium | High | Limited |
| Advantages | -Direct measurement -Numeric value obtained -More rapid that the Group 1 -No dilution | -Quantitative measurement of the viral titer -Flexibility and versatility | -Direct quantitative measurement of the viral titer -Direct observation of viral plaques | -Direct quantitative measurement of the viral titer -Direct observation of viral plaques | -Rapid and specific | -Sensitive  -Allows specific detection of antigens | -Simple and rapid | -Rapid -Sensitive and specific | -Very short analysis time | -Direct visualization (morphology, structure, size, interaction between virus and cells) |
| Disadvantages | -Do not measure infectivity -No standard protocol -Sensitive to light | -Time-consuming -Requires serial dilutions -Limited to certain types of viruses, can be difficult for strains that do not form clear, distinct plaques -Subjective observations | -Time consuming -Requires serial dilutions -Limited to certain types of viruses, can be difficult for strains that do not form clear, distinct plaques | -Time consuming -Requires serial dilutions | -Do not measure infectious particles | -Do not measure infectivity -Dependence on high quality reagents -Possibility of false positives | -Do not measure infectivity -Require fresh Red Blood Cells (RBCs) for good reproducibility -Limited to a certain type of viruses -Low sensitivity | -Do not measure infectivity -Dependence on high quality reagents -Possibility of false positives | -Expensive -Specific flow Cytometric equipment (expensive apparatus) -Complexity | -Do not quantify infectivity -No distinction between infectious and non-infectious particles |
| References | This work | (Lei et al., 2021; Reed & Muench, 1938) | (Baer & Kehn-Hall, 2014; Mendoza et al., 2020) | (Truant,’ et al., 1983) | (Kralik & Ricchi, 2017) | (Ma et al., 2011) | (Killian, 2014) | (Atiya-Nasagi et al., 2022) | (Lippé, 2018; Zamora & Aguilar, 2018) | (Richert-Pöggeler et al., 2019) |

**
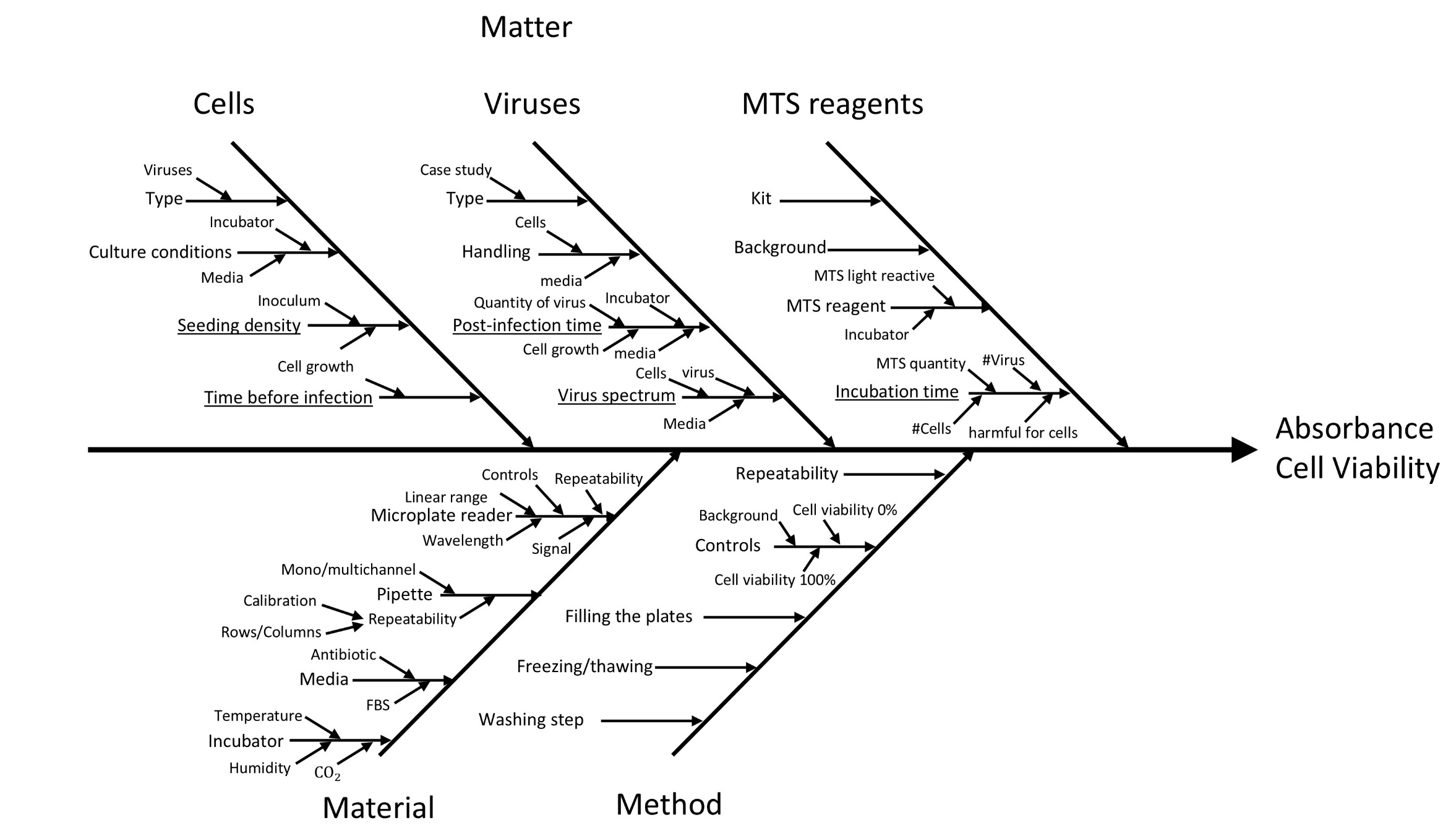
**

Figure S1: Ishikawa diagram summarizing the useful causes and effects to be established for optimizing the MTS assay. It consists of several branches: material, equipment and the method used.


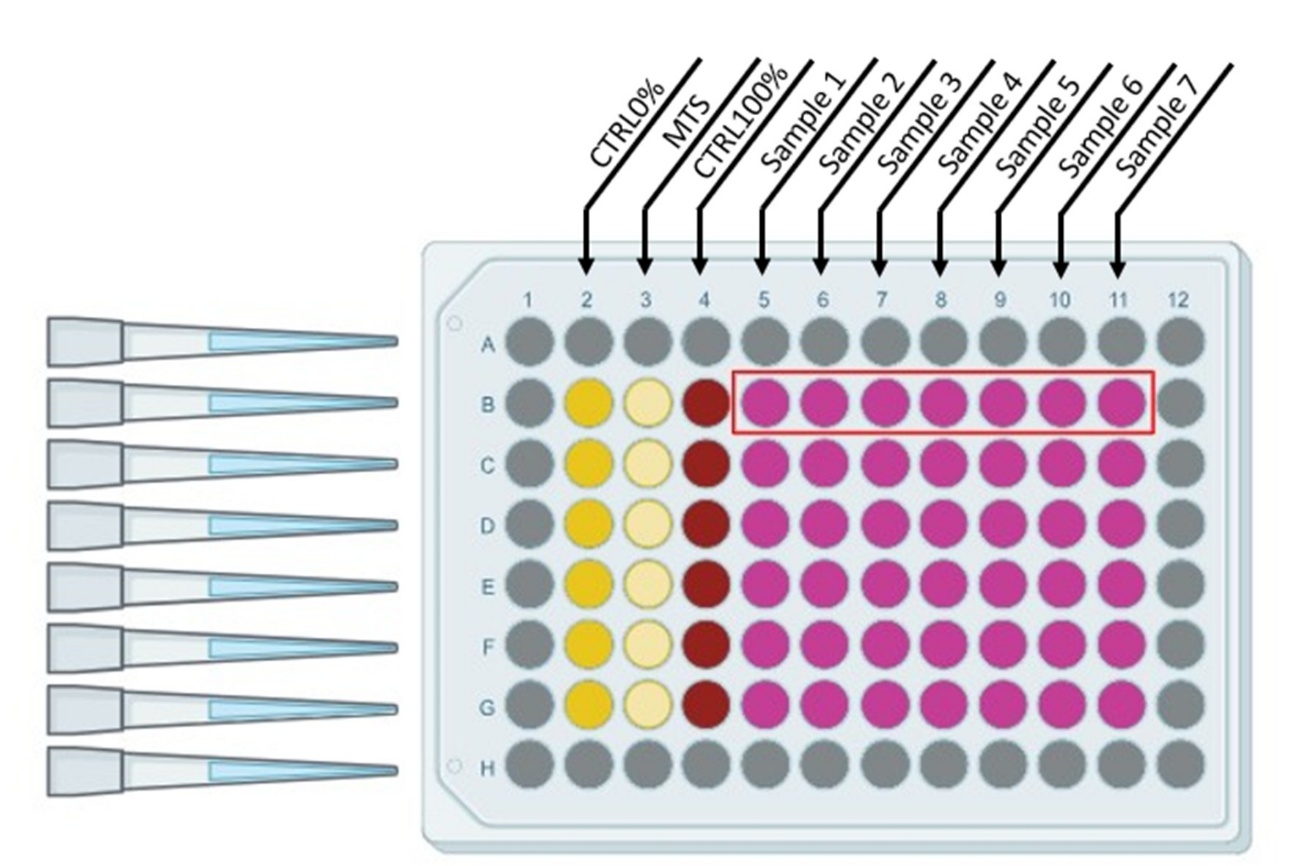


| Color | Wells | Description |
| --- | --- | --- |
| Dark yellow | B2-G2 | These wells are used as positive controls ($A_{CTRL0\%}$). They are seeded and placed in contact with a detergent before MTS reading. |
| Light yellow | B3-G3 | These wells are used to determine the background ($A_{MTS})$ and are only filled with a mixture of MTS reagent and culture medium. By comparison with the outermost wells (grey color), this method can be used to assess any internal measurement gradients due to instrument malfunction, culture plate variability or problems with the MTS reagents. |
| Dark red | B4-G4 | These wells serve as negative controls where the cells are not infected by the viruses ($A_{CTRL100\%}$). These same wells can also be used to study variations in the number of cells seeded with the multichannel pipette. They can be used to detect technical problems with the pipette or absorbance measurement. |
| Wells inside red squartes | B5-B11 | These wells assess the variance of multichannel pipetting in cell seeding density. Vehicle-treated cells are seeded at different ejection stages.  Any variation in absorbance may indicate handling problems when seeding the cells or when reading the absorbance. The value of the wells must be compared with those of the negative controls (B4-G4). |
| Grey | A1-A12, B1-G1, B12-G12 and H1-H12 | These wells must contain only culture medium from cell seeding to MTS reading. This avoids edge effects during multiple incubations, such as evaporation from the wells. It has been shown that the absorbance of these wells after reading was different from that of the central wells (Patel et al., 2005). In comparison with column 3, the addition of MTS reagent to these wells enables the reading and pipetting performance to be evaluated. |
| Purple | C5-G5  C6-G6  C7-G7  C8-G8  C9-G9  C10-G10  C11-G11 | These wells are used to titrate unknown viral solutions. 7 titrations are possible per plaque, associated with each column with 5 replicates. |

Figure S2: Image illustrating the layout of a 96-well plate for determining the titration of a virus solution by the tetrazolium method. The arrows represent the filling by column. The plate is filled column by column using a multichannel pipette. The role of each well is explained in the table.


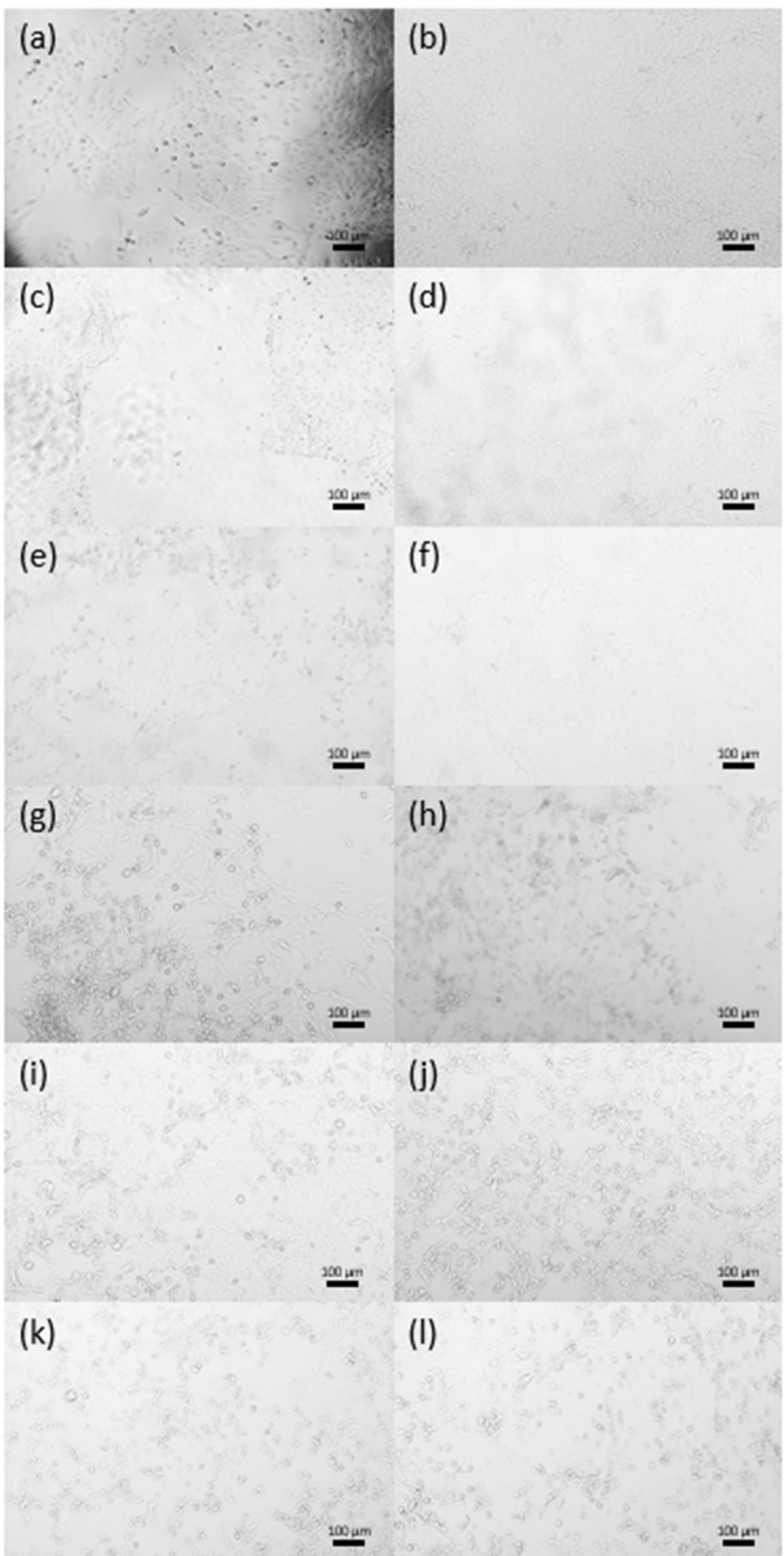


Figure S3: Optical micrographs taken 24h post-seeding of PRCV and ST Cells, (a) before infection, and, 72h post-infection (b) mock-infected cells (negative control), (c) cells with the triton X-100 compounds (positive control). (d-l) are images taken 72h post-infection infected with cells 50, 100, 200, 1,000, 1,500, 2,000, 3,000 and 5,000 $\mathrm{TCID}_{50}/ml$, respectively, showing the cytopathic effect of viruses on the cells. It is noticeable that as the viral dose increases, the cytopathic effect on the cell layer becomes more pronounced. The infected cells exhibit morphological changes, ranging from cell lysis to nearly the entire cell monolayer detaching from the plate.

**Annexe 1: Modeling of Viral Dilution: A Logistic Approach**

The cell viability curve as a function of virus load can be modeled using a logistic equation described as follows.

$$CV\left( \% \right)=A_{2}+\frac{\left( A_{1}-A_{2} \right)}{\left( 1+\left( \frac{x}{x_{0}} \right)^{p} \right)}$$

where

- CV: represents the viral solution, expressed as a percentage (%). This value is derived from absorbance measurements ($A_{MTS}$) after subtracting the background measurement. ($A_{CTRL0\%})$ indicates complete virus toxicity towards cells (positive control), and ($A_{CTRL100\%}$) represents total viability with the absorbance value of mock-infected cells.
- *x:* represents viral concentration, often expressed as the logarithm of the viral titer
- *A1:* represents the maximum asymptotic cell viability, i.e., the viability for the mock-infected cells
- *A2:* represents the minimum asymptotic cell viability, i.e., the viability close to the triton or at the very high viral dose
- *x0:* represents the viral at which the cell viability is halfway between A1 and A2.
- *p:* represents the slope of the logistic curve.


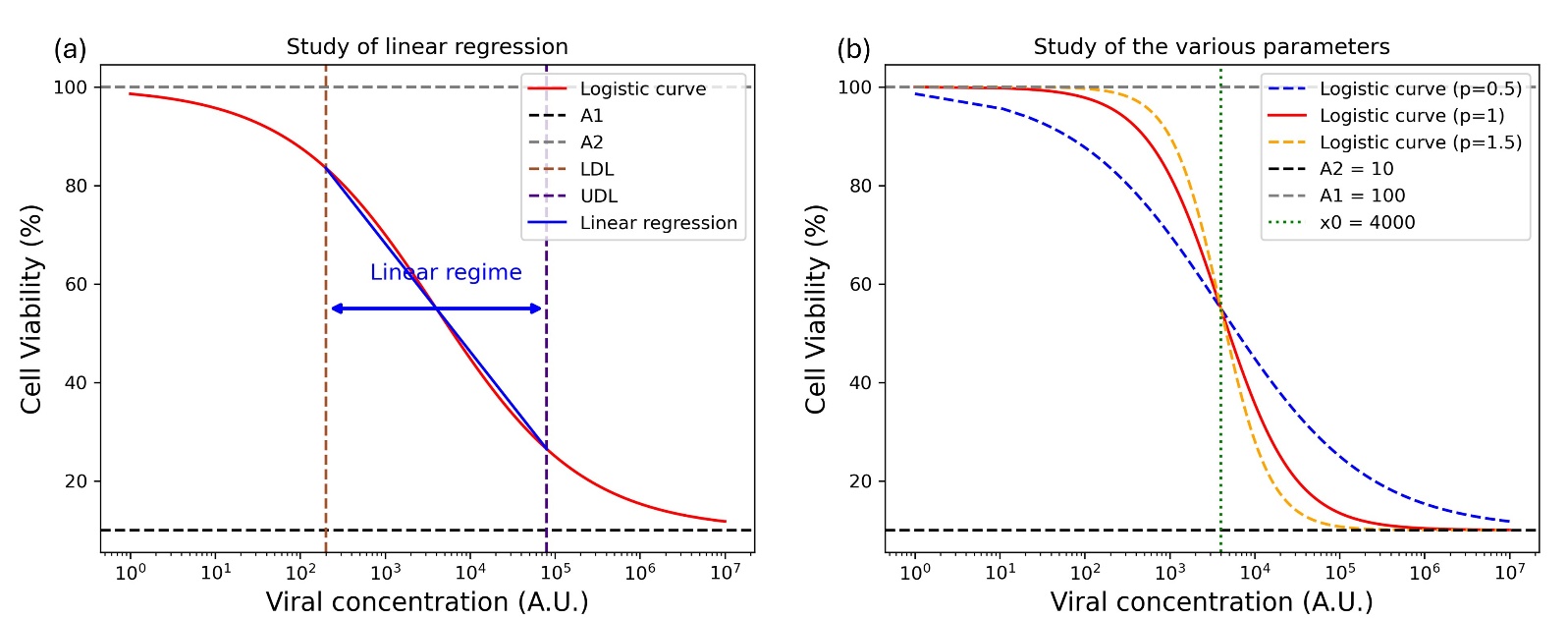


Figure S4: Modeling of cell viability curve as a function of virus load: (a) illustrates the relationship between cell viability and viral concentration, while (b) describes the different parameters (A1, A2, x0, and p).

**Curve interpretation:**The sigmoid curve obtained by plotting cell viability (y) against viral concentration (x) exhibits three distinct phases:

**Initial phase:** At low concentrations (x low), cell viability remains close to the maximum value (A2).

**Transition phase:** At intermediate concentrations (x around x0), cell viability decreases rapidly in a nearly linear manner. This linear region enables the establishment of calibration, allowing the conversion of cell viability values obtained from absorbance readings into viral titer (in TCID50/ml or PFU/ml).

**Plateau phase:** At high concentrations (x high), infectivity approaches the maximum level (A1), which is close to that observed with Triton.

**Supplementary information: Optimization of cell and viral cultures for PRCV and BoHV-1**

The protocol outlined below is applied for the Porcine Respiratory Coronavirus (PRCV) and the Bovine alpha herpesvirus (BoHV-1), and hosts cells, Swine Testicular (ST) cells and Madin-Darby Bovine Kidney (MDBK ) cells, respectively. This process comprises 3 main steps: seeding the cells into a 96-well plate, infecting these cells with virus from the desired titration solution, and subsequently, assessing using the MTS reagent. Some cell culture or viral parameters may vary depending on the host species or virus used. This is particularly the case for the 5 parameters: (i) the cellular concentration to be seeded per well, (ii) the cell growth time between seeding and infection, (iii) the viral spectrum that infects these cells, (iv) the post-infection time required for the viruses to cause cytopathic effects on these cells, and finally, (v) the time required for the conversion of MTS to formazan to be significant.

**Seeding of Cells (Day 0)**

1. The ST Cells (ATCC CRL-1746) or MDBK (NBL-1) cells are cultured as described by the ATCC protocol. These cells are cultured in Eagle’s Minimum Essential Medium (EMEM) containing 10% Fetal Bovine Serum (FBS), L-glutamine (292 µg/L), 100 U/ml Penicillin and 100µg/mL Streptomycin, 1 mM sodium pyruvate (or 1mM MEM Eagle non-essential amino acids – NEAA for MDBK cells) and under cell growth conditions (37°C, 95% RH and 5% CO_2_). Subculturing is necessary when adherent cells reach full confluence in the culture flask.
2. 24h before tittering, a cell suspension is taken at the desired concentration. To that end, it is necessary to determine the cell concentration to seed into each well of the 96-well plate by cell counting, either by manually using an hemocytometer or by using an automated cell counter. The plate is filled, column per column, with a multichannel, according to the plate layout proposed in figure S2, or, according to the previously established manipulation plan. The volume and the cell concentration are 100 µl/well and 60 000 cells/ml for ST Cells and 100 000 cells/ml for MDBK cells.
3. The cells are incubated at 37°C for 24h in 5% CO_2_.

**Infection (Day 1)**

1. After removing the medium, transfer the viral content into the plate, allocating it from B5 to G11, with each condition occupying a separate column, following the prearranged plate layout (Figure S2). The medium used involved 100µl for each well. It included EMEM with 1% Fetal Bovine Serum (FBS), L-glutamine (292 µg/L), 100 U/ml Penicillin, 100 µg/ml Streptomycin, and 1 mM sodium pyruvate or 1mM MEM Eagle non-essential amino acids – NEAA for MDBK cells.
2. Remember to add 100 µl of medium to the uninfected wells, serving as controls (in columns 2 and 4). At this stage, column 3 can be left empty, as it functions as a control for background measurement.
3. After 2 hours, 100 µl was adding in each well, and cells were incubated at 37°C in 5% CO_2_for 72 hours for ST cells and 48h MDBK cells.

**MTS reading (Day 4)**

1. A solution containing a mixture of MTS and EMEM medium is prepared in a ratio of 1:6 (20µl of MTS and 100 µl of media). For a complete 96-well plate, a volume of 11.52ml of this solution is required, which is 1.92ml of MTS reagent and 9.6ml of media. CAUTION: it is essential to avoid light exposition of the sensitive MTS reagent.
2. Remove the cells contained in the negative control wells with detergent (e.g. Triton X-100).
3. After removing the medium in each well, this mixture is added to each well using a pipette, filling the plates by columns. Again, this step must be carried out away from the light.
4. The 96-well plate is incubated at 37°C for 1h30 in 5% CO_2_. During this incubation time, the MTS reagent is transformed into formazan, and the color of media in each well changes from golden yellow to purple. This color change provides an initial indication of the result.
5. Using a plate spectrophotometer, the absorbance measurement is determined for each well at 490nm. CAUTION: it is important that each well has the same volume and does not contain bubbles or other elements that could bias the measurement.

**Data analysis (Day 4)**

1. Based on the obtained absolute absorbance values, determine the means and standard deviations of the test samples as well as those of the controls.
2. Normalize these results relative to controls with 100% viability to obtain cell viability as a percentage. The formula below enable the percentage calculation :
3. $Cell viability \left( \% \right)= \frac{A_{tested}-A_{MTS}}{A_{CTRL100\%}-A_{MTS}}\times100$
4. Determine the analysis parameters specific to the high-throughput screening assay to determine test effectiveness: Z factor, signal-to-noise ratio (S/N), and signal-to-background (S/B), as well as RSD(%) for each tested sample.

Table S2: List of reagents and their product numbers

| **Reagents** | **Corporation** | **Products number** |
| --- | --- | --- |
| EMEM | Thermo Fischer | 41090028 |
| Trypsine EDTA | Thermo Fischer | 25300054 |
| PBS | Thermo Fischer | 10010023 |
| FBS | Thermo Fischer | 10270-106 |
| Penicillin-Streptomycin | Gibco | 15140122 |
| Sodium pyruvate | Lonza | BE13-115E |
| MEM NEAA | Gibco | 11140-035 |
| 96-well plate | Corning | CLS3596 |
| MTS solution | Promega | G3581 |
| Triton | / | 4744977001 |
| Microplate Reader | SpectraMax iD3 (Molecular Devices, San Jose, CA, USA, RRID:SCR_023920) | / |

## References

Atiya-Nasagi, Y., Milrot, E., Makdasi, E., Schuster, O., Shmaya, S., Simon, I., Ben-Shmuel, A., Beth-Din, A., Weiss, S., & Laskar, O. (2022). Development of an immunofluorescence assay for detection of SARS-CoV-2. *Archives of Virology*, *167*(4), 1041–1049. https://doi.org/10.1007/s00705-022-05392-z

Baer, A., & Kehn-Hall, K. (2014). Viral concentration determination through plaque assays: Using traditional and novel overlay systems. *Journal of Visualized Experiments*, *93*. https://doi.org/10.3791/52065

Killian, M. L. (2014). Hemagglutination assay for influenza virus. *Methods in Molecular Biology*, *1161*, 3–9. https://doi.org/10.1007/978-1-4939-0758-8_1

Kralik, P., & Ricchi, M. (2017). A basic guide to real time PCR in microbial diagnostics: Definitions, parameters, and everything. In *Frontiers in Microbiology* (Vol. 8, Issue FEB). Frontiers Research Foundation. https://doi.org/10.3389/fmicb.2017.00108

Lei, C., Yang, J., Hu, J., & Sun, X. (2021). On the Calculation of TCID50 for Quantitation of Virus Infectivity. In *Virologica Sinica* (Vol. 36, Issue 1, pp. 141–144). Science Press. https://doi.org/10.1007/s12250-020-00230-5

Lippé, R. (2018). *Flow Virometry: a Powerful Tool To Functionally Characterize Viruses*. https://doi.org/10

Ma, L. N., Zhang, J., Chen, H. T., Zhou, J. H., Ding, Y. Z., & Liu, Y. S. (2011). An overview on ELISA techniques for FMD. In *Virology Journal* (Vol. 8). https://doi.org/10.1186/1743-422X-8-419

Mendoza, E. J., Manguiat, K., Wood, H., & Drebot, M. (2020). Two Detailed Plaque Assay Protocols for the Quantification of Infectious SARS-CoV-2. *Current Protocols in Microbiology*, *57*(1). https://doi.org/10.1002/cpmc.105

Patel, M. I., Tuckerman, R., & Dong, Q. (2005). A pitfall of the 3-(4,5-dimethylthiazol-2-yl)-5(3-carboxymethonyphenol)-2- (4-sulfophenyl)-2H-tetrazolium (MTS) assay due to evaporation in wells on the edge of a 96 well plate. *Biotechnology Letters*, *27*(11), 805–808. https://doi.org/10.1007/s10529-005-5803-x

Reed, L. J., & Muench, H. (1938). A SIMPLE METHOD OF ESTIMATING FIFTY PER CENT ENDPOINTS l ’ 2. In *THE AMERICAN JOURNAL OF HYGIENE* (Vol. 27, Issue 3). https://academic.oup.com/aje/article-abstract/27/3/493/99616

Richert-Pöggeler, K. R., Franzke, K., Hipp, K., & Kleespies, R. G. (2019). Electron microscopy methods for virus diagnosis and high resolution analysis of viruses. In *Frontiers in Microbiology* (Vol. 10, Issue JAN). Frontiers Media S.A. https://doi.org/10.3389/fmicb.2018.03255

Truant,’, A. L., Regnery, R. L., & Kiley2, M. P. (1983). Development of an Immunofluorescence Focus Assay for Ebola Virus. In *JOURNAL OF CLINICAL MICROBIOLOGY* (Vol. 18, Issue 2).

Zamora, J. L. R., & Aguilar, H. C. (2018). Flow virometry as a tool to study viruses. In *Methods* (Vols. 134–135, pp. 87–97). Academic Press Inc. https://doi.org/10.1016/j.ymeth.2017.12.011
